# Effect of Alirocumab on Stroke in ODYSSEY OUTCOMES

**DOI:** 10.1161/CIRCULATIONAHA.119.043826

**Published:** 2019-11-11

**Authors:** J. Wouter Jukema, Laurien E. Zijlstra, Deepak L. Bhatt, Vera A. Bittner, Rafael Diaz, Heinz Drexel, Shaun G. Goodman, Yong-Un Kim, Robert Pordy, Željko Reiner, Matthew T. Roe, Hung-Fat Tse, Pablo Carlos Montenegro Valdovinos, Harvey D. White, Andreas M. Zeiher, Michael Szarek, Gregory G. Schwartz, Philippe Gabriel Steg

**Affiliations:** 1 Department of Cardiology, Leiden University Medical Center, The Netherlands (J.W.J., L.E.Z.).; 2 Brigham and Women’s Hospital Heart & Vascular Center and Harvard Medical School, Boston, Massachusetts (D.L.B.).; 3 Division of Cardiovascular Disease, University of Alabama at Birmingham (V.A.B.).; 4 Estudios Cardiológicos Latinoamérica, Instituto Cardiovascular de Rosario, Rosario, Argentina (R.D.).; 5 Landeskrankenhaus Feldkirch, Austria (H.D.).; 6 Division of Angiology, Swiss Cardiovascular Center, University of Bern, Switzerland (H.D.).; 7 Canadian VIGOUR Centre, University of Alberta, Edmonton, Canada (S.G.G.).; 8 St. Michael’s Hospital, University of Toronto, Ontario, Canada (S.G.G.).; 9 Sanofi, Paris, France (Y-U.K.).; 10 Regeneron Pharmaceuticals Inc., Tarrytown, New York (R.P.).; 11 University Hospital Center Zagreb, School of Medicine, University of Zagreb, Croatia (Z.R.).; 12 Duke Clinical Research Institute, Duke University Medical Center, Durham, North Carolina (M.T.R.).; 13 Queen Mary Hospital, The University of Hong Kong (H-F.T.).; 14 Unidad de Diagnostico Cardiologico, Guatemala City, Guatemala (P.C.M.V.).; 15 Hospital General San Juan de Dios, Guatemala City, Guatemala (P.C.M.V.).; 16 Green Lane Cardiovascular Services, Auckland City Hospital, Auckland, New Zealand (H.D.W.).; 17 Department of Medicine III, Goethe University, Frankfurt am Main, Germany (A.M.Z.).; 18 State University of New York, Downstate School of Public Health, Brooklyn (M.S.).; 19 Division of Cardiology, University of Colorado School of Medicine, Aurora (G.G.S.).; 20 Assistance Publique-Hôpitaux de Paris, Hôpital Bichat, Université de Paris, FACT (French Alliance for Cardiovascular Trials), INSERM U1148, Paris (P.G.S.).; 21 National Heart and Lung Institute, Imperial College, Royal Brompton Hospital, London, United Kingdom (P.G.S.).

**Keywords:** acute coronary syndrome, cerebrovascular disorders, lipoprotein, LDL, stroke

## Abstract

**Background:**

Lowering of atherogenic lipoproteins, including low-density lipoprotein cholesterol (LDL-C), reduces the risk of ischemic stroke. However, concerns have been raised about very low LDL-C levels and a potential increased risk of hemorrhagic stroke. ODYSSEY OUTCOMES compared the PCSK9 inhibitor alirocumab with placebo in 18 924 patients with recent acute coronary syndrome and elevated atherogenic lipoproteins, despite intensive statin therapy, targeting LDL-C levels of 25 to 50 mg/dL and avoiding sustained LDL-C <15 mg/dL. This prespecified analysis was designed to assess the effect of alirocumab on ischemic and hemorrhagic stroke. We hypothesized that for patients treated with alirocumab there would be a reduction in risk of ischemic stroke without increasing hemorrhagic stroke, irrespective of baseline LDL-C and of history of cerebrovascular disease.

**Methods:**

Patients were randomized to alirocumab or placebo 1 to 12 months after acute coronary syndrome. The risk of nonfatal or fatal ischemic or hemorrhagic stroke was evaluated, stratified by baseline LDL-C concentration and history of cerebrovascular disease. A potential association of very low achieved LDL-C with alirocumab treatment at month 4 and subsequent hemorrhagic stroke was assessed.

**Results:**

Median follow-up was 2.8 years. In total, 263 ischemic and 33 hemorrhagic strokes occurred. Alirocumab reduced the risk of any stroke (HR, 0.72 [95% CI, 0.57−0.91]) and ischemic stroke (HR, 0.73 [95% CI, 0.57−0.93]) without increasing hemorrhagic stroke (HR, 0.83 [95% CI, 0.42−1.65]). In total, 7164 (37.9%), 6128 (32.4%), and 5629 (29.7%) patients had a baseline LDL-C of <80, 80 to 100, and >100 mg/dL, respectively. The treatment effect on stroke appeared numerically greater for patients with higher baseline LDL-C, but there was no formal evidence of heterogeneity (*P*_interaction_=0.31). The effect of alirocumab on stroke was similar among 944 patients (5.0%) with a history of previous cerebrovascular disease and among those without a history of cerebrovascular disease (*P*_interaction_=0.37). There was no apparent adverse relation between lower achieved LDL-C and incidence of hemorrhagic stroke in the alirocumab group.

**Conclusions:**

In patients with recent acute coronary syndrome and dyslipidemia despite intensive statin therapy, alirocumab decreased the risk of stroke, irrespective of baseline LDL-C and history of cerebrovascular disease, over a median follow-up of 2.8 years. Furthermore, risk of hemorrhagic stroke did not depend on achieved LDL-C levels within the alirocumab group.

**Clinical Trial Registration:**

URL: https://www.clinicaltrials.gov. Unique identifier: NCT01663402.

## Abstract 2

Supplemental Digital Content is available in the text.

**Clinical Perspective**

**What Is New?**

- In a randomized, double-blind trial in 18 924 patients with acute coronary syndrome and elevated atherogenic lipoproteins despite intensive statin treatment, PCSK9 (proprotein convertase subtilisin−kexin type 9) inhibition with alirocumab significantly decreased the risk of ischemic stroke, without increasing hemorrhagic stroke.
- This effect did not depend on baseline low-density lipoprotein cholesterol or history of cerebrovascular disease.
- Furthermore, the present findings indicate that risk of hemorrhagic stroke did not depend on achieved low-density lipoprotein cholesterol levels in the alirocumab group.

**What Are the Clinical Implications?**

- Alirocumab added to intensive statin therapy provides an opportunity to lower low-density lipoprotein cholesterol to levels not previously achievable in most patients with statins and/or ezetimibe.
- Lowering of low-density lipoprotein cholesterol to very low levels reduces the risk of ischemic stroke without increasing hemorrhagic stroke.

**Editorial, see p 2063**

Lowering of atherogenic lipoproteins with statin treatment reduces the risk of first or recurrent stroke,^1–3^ and the benefit has been shown by the Cholesterol Treatment Trialist meta-analysis to be directly proportional to the degree of absolute lowering of low-density lipoprotein cholesterol (LDL-C).^4^ Accordingly, international guidelines recommend statin treatment for patients at high cardiovascular risk or with established cardiovascular disease with or without a history of cerebrovascular disease, to prevent major cardiovascular events, including ischemic stroke.^5,6^

Although some data have raised a potential association of very low LDL-C levels and risk of hemorrhagic stroke,^7,8^ the decrease in ischemic stroke outweighed the potential increase in hemorrhagic stroke.^4,9^ The advent of inhibitors of PCSK9 (proprotein convertase subtilisin−kexin type 9) provided an opportunity to lower LDL-C to levels not previously achievable in most patients with statins and/or ezetimibe. Two large cardiovascular outcomes trials have compared the effect of a fully human PCSK9 inhibitor with placebo on the risk of stroke in patients with atherosclerotic cardiovascular disease and elevated atherogenic lipoproteins despite background statin treatment.^10,11^ In both trials, treatment with the PCSK9 inhibitor lowered LDL-C by more than 50% below the statin-treated baseline. The FOURIER trial (Further Cardiovascular Outcomes Research With PCSK9 Inhibition in Patients With Elevated Risk)^10^ compared evolocumab with placebo in patients with established, stable, atherosclerotic cardiovascular disease. Evolocumab treatment reduced the risk of ischemic stroke without a significant effect on hemorrhagic stroke. The ODYSSEY OUTCOMES trial (Evaluation of Cardiovascular Outcomes After an Acute Coronary Syndrome During Treatment With Alirocumab)^11^ compared alirocumab with placebo in 18 924 patients with recent acute coronary syndrome (ACS) and showed a reduction in major adverse cardiovascular events with alirocumab compared with placebo. This prespecified analysis was designed to assess the effect of alirocumab on ischemic and hemorrhagic stroke. We hypothesized that for patients treated with alirocumab, there would be a reduction in risk of ischemic stroke without increasing hemorrhagic stroke, irrespective of baseline LDL-C and of history of cerebrovascular disease.

## Methods

The data that support the findings of this study are available from the corresponding author upon reasonable request.

Details of the study design^12^ and primary efficacy and safety results^11^ have been published. In brief, ODYSSEY OUTCOMES was a multicenter, double-blind, placebo-controlled trial in 18 924 patients at least 40 years of age who provided written informed consent and had been hospitalized with an ACS (defined as myocardial infarction or unstable angina) 1 to 12 months before randomization. Qualifying patients had a level of LDL-C ≥70 mg/dL (1.81 mmol/L), or non−high-density lipoprotein cholesterol (non–HDL-C) ≥100 mg/dL (2.59 mmol/L), or apolipoprotein B ≥80 mg/dL (0.8 mmol/L), measured after a minimum of 2 weeks of stable treatment with atorvastatin 40 to 80 mg daily, rosuvastatin 20 to 40 mg daily, or the maximum tolerated dose of either statin (including no statin in case of documented intolerance). All sites obtained institutional review board approval as per local and national guidelines.

Patients were randomly assigned in a 1:1 ratio stratified by country to receive treatment with alirocumab 75 mg subcutaneously every 2 weeks or matching placebo. In case of a persistent LDL-C ≥50 mg/dL, the dose of alirocumab was up-titrated to 150 mg/dL. When 2 consecutive measurements of LDL-C <25 mg/dL were identified, the alirocumab dose was reduced to 75 mg (if measurements were made on the 150-mg dose), and safety was monitored by an independent physician blinded to treatment allocation. In case of two consecutive measurements of LDL-C <15 mg/dL on alirocumab 75 mg, alirocumab was discontinued with blinded substitution of placebo for the remainder of the trial. The protocol did not specify any change to the background statin dose.

Patients were compared based on three prespecified categories of baseline LDL-C (ie, <80 mg/dL, 80 to 100 mg/dL, ≥100 mg/dL). We compared the effect of alirocumab on stroke among patients with or without a history of cerebrovascular disease, defined as a history of carotid endarterectomy, carotid stenting, previous stroke, or transient ischemic attack. A multivariable prediction model of stroke risk was performed. Last, a subgroup analysis was performed based on the treat-to-target design of the trial, where patients assigned to alirocumab treatment were classified in the following categories on the basis of their achieved LDL-C value at month 4: below target range (<25 mg/dL), within target range (25 to <50 mg/dL), above target range (50 to <70 mg/dL), and very above target range (≥70 mg/dL). In this analysis, the incidence of hemorrhagic stroke after month 4 was summarized among patients in each of these achieved LDL-C categories.

### End Points

End points were classified as fatal or nonfatal ischemic or hemorrhagic stroke, adjudicated by physicians who were unaware of the study treatment group assignments. As part of the original analysis conventions, an ischemic or unclassified stroke that was followed by a death within 30 days with a cause of ischemic or undetermined stroke was considered a fatal ischemic stroke, with an event date of the initial event. For the purposes of the current report, this convention was applied for hemorrhagic strokes (ie, a hemorrhagic stroke that was followed by a death within 30 days was considered a fatal hemorrhagic stroke, with an event date of the initial event). In addition, 9 nonfatal strokes with an unclassified cause were grouped with nonfatal ischemic strokes, as prespecified in the design of the study.

### Statistical Considerations

Analyses of clinical outcomes and LDL-C levels were performed according to the intention-to-treat principle, including all patients, events, and measurements from randomization to the study end date (November 11, 2017). Hazard ratios (HR) and 95% CIs were estimated by Cox proportional hazards models, stratified by geographic region; *P* values were determined using stratified log-rank tests. End point rates were based on observed incidences. The treatment proportional hazards assumption for each type of stroke (any, ischemic, hemorrhagic) was assessed by a Kolmogorov-type supremum test. A multivariable model was performed to predict all-cause stroke with stepwise selection, using *P*=0.05 for entry or exit. Prespecified candidate variables were age category, sex, race, region, index event, lipid-lowering therapy at randomization, LDL-C, HDL-C, lipoprotein(a), body mass index, systolic blood pressure, glomerular filtration rate, diabetes, hypertension, myocardial infarction, cerebrovascular disease, malignant disease, percutaneous coronary intervention, chronic obstructive pulmonary disease, coronary artery bypass grafting, peripheral artery disease, chronic heart failure, venous thromboembolism, atrial fibrillation, current smoker, revascularization for index event, oral adenosine diphosphate receptor antagonist, oral anticoagulant, and alirocumab treatment. Relationships between categories of achieved month-4 LDL-C and subsequent hemorrhagic stroke in the alirocumab group were summarized by descriptive statistics. Analyses were performed in SAS 9.4 and S+ 8.2.

## Results

Of 18 924 randomized patients, 9462 were assigned to the alirocumab group and 9462 to the placebo group, with a median (quartile 1, quartile 3) follow-up of 2.8 (2.3, 3.4) years. There were no major differences in baseline characteristics between the alirocumab group and the placebo group.^11^ At baseline, there were 944 patients (5.0%) with a history of cerebrovascular disease and 17 980 (95.0%) without a history of cerebrovascular disease.

Table 1 summarizes the baseline characteristics of patients with or without a history of cerebrovascular disease. Compared with patients without a history of cerebrovascular disease, those with cerebrovascular disease were older (median age, 63 vs 58 years) and included more women (31.9% vs 24.8%). Of all patients with cerebrovascular disease, 611 (64.7%) had a history of stroke. Furthermore, compared with patients without a history of cerebrovascular disease, those with cerebrovascular disease had a higher systolic blood pressure and more often had comorbidities, including a history of diabetes, hypertension, myocardial infarction, atrial fibrillation, peripheral artery disease, venous thromboembolism, chronic obstructive pulmonary disease, heart failure, malignant disease, percutaneous coronary intervention, coronary artery bypass grafting, and a glomerular filtration rate <60 mL/min/1.73m^2^). Median (quartile 1, quartile 3) baseline LDL-C was 91 (76 110) mg/dL in patients with cerebrovascular disease versus 86 (73 104) mg/dL in those without cerebrovascular disease.

**Table 1.**
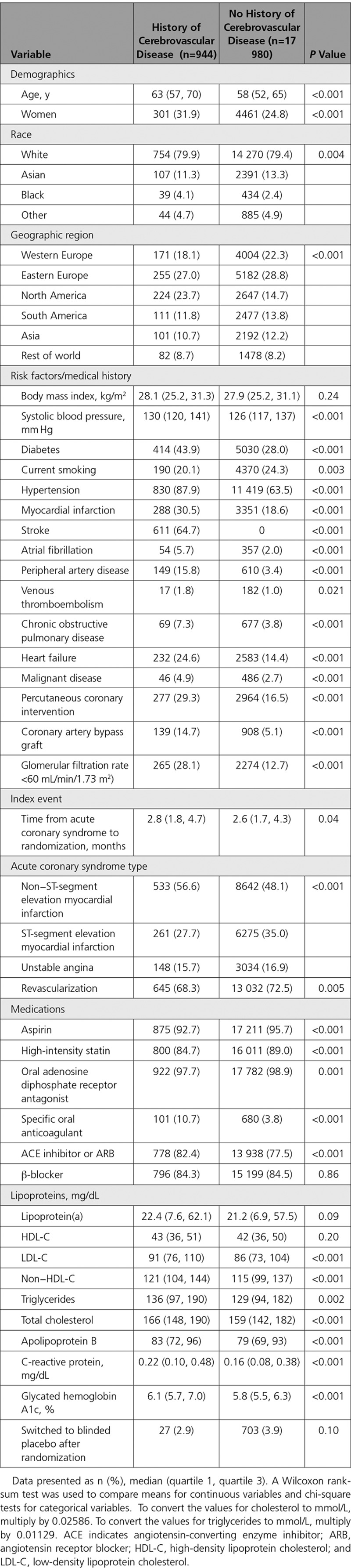
Baseline Characteristics, by History of Cerebrovascular Disease

**Figure 1.**
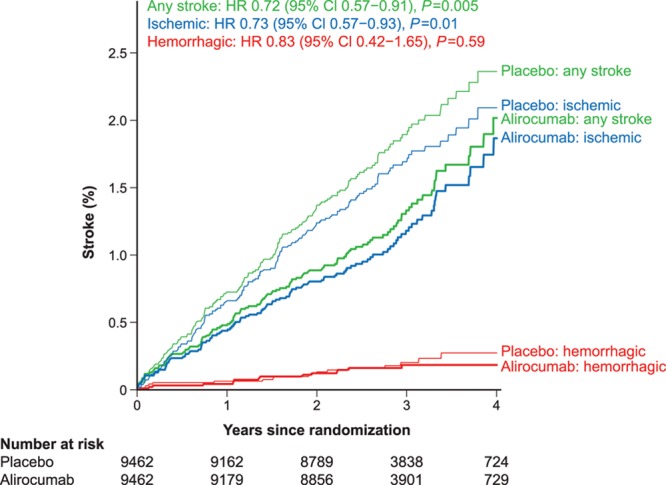
**Kaplan-Meier curves for any stroke, ischemic stroke and hemorrhagic stroke.** CI indicates confidence interval; and HR, hazard ratio.

The Kaplan-Meier curves for any stroke, ischemic stroke, and hemorrhagic stroke are shown in Figure 1. In total, 263 ischemic strokes and 33 hemorrhagic strokes occurred. Of the 33 hemorrhagic strokes, 25 occurred in the safety population during the treatment-emergent adverse event reporting period,^11^ and 8 were captured in the intention-to-treat analysis. Alirocumab reduced the risk of any stroke (HR, 0.72 [95% CI, 0.57–0.91]) and ischemic stroke (HR, 0.73 [95% CI, 0.57–0.93]) without increasing hemorrhagic stroke (HR, 0.83 [95% CI, 0.42–1.65]). There was no evidence of nonproportionality in the treatment effects (supremum test *P*=0.56, 0.35, and 0.47 for any, ischemic, and hemorrhagic, respectively).

**Figure 2.**
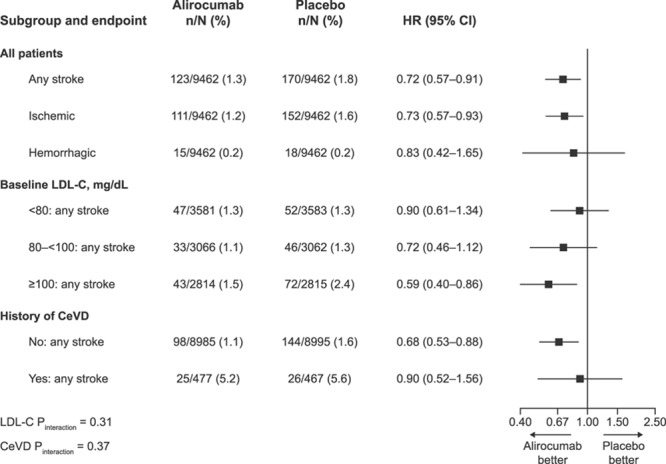
**Stroke by history of cerebrovascular disease and baseline LDL-C category.** LDL-C indicates low-density lipoprotein cholesterol; and HR, hazard ratio.

Figure 2 shows the HRs for stroke by baseline LDL-C category and history of cerebrovascular disease. In total, 7164 (37.9%) patients had a baseline LDL-C <80 mg/dL, 6128 (32.4%) had a value of 80 to 100 mg/dL, and 5629 (29.7%) had a value >100 mg/dL. The treatment effect appeared numerically greater for patients with higher baseline LDL-C, but there was no formal evidence of treatment effect heterogeneity (*P*_interaction_=0.31). An exploratory analysis was performed in which baseline LDL-C was categorized dichotomously (<100 mg/dL and ≥100 mg/dL), which also found no formal evidence of treatment effect heterogeneity (*P*_interaction_=0.18). Similarly, the effect of alirocumab on stroke appeared consistent regardless of the presence (n=944 patients [5.0%]) or absence of a history of cerebrovascular disease, (*P*_interaction_=0.37).

The multivariable baseline predictors of any stroke are shown in Table 2. History of cerebrovascular disease was the strongest predictor, with a HR of 2.469 (95% CI, 1.792–3.401; *P*<0.0001). In addition, glomerular filtration rate <60 mL/min/1.73 m^2^), diabetes, heart failure, myocardial infarction, oral anticoagulants, current smoking and peripheral artery disease, and increasing age, systolic blood pressure, and LDL-C were associated with an increased risk of all-cause stroke (all *P*<0.05). Alirocumab had the strongest negative association with stroke (HR, 0.712 [95% CI, 0.564–0.898]; *P*=0.0041). Per 1-mg/dL increment, HDL-C also had a negative association with stroke (HR, 0.989 [95% CI, 0.978–1.000]; *P*=0.0476).

**Table 2.**
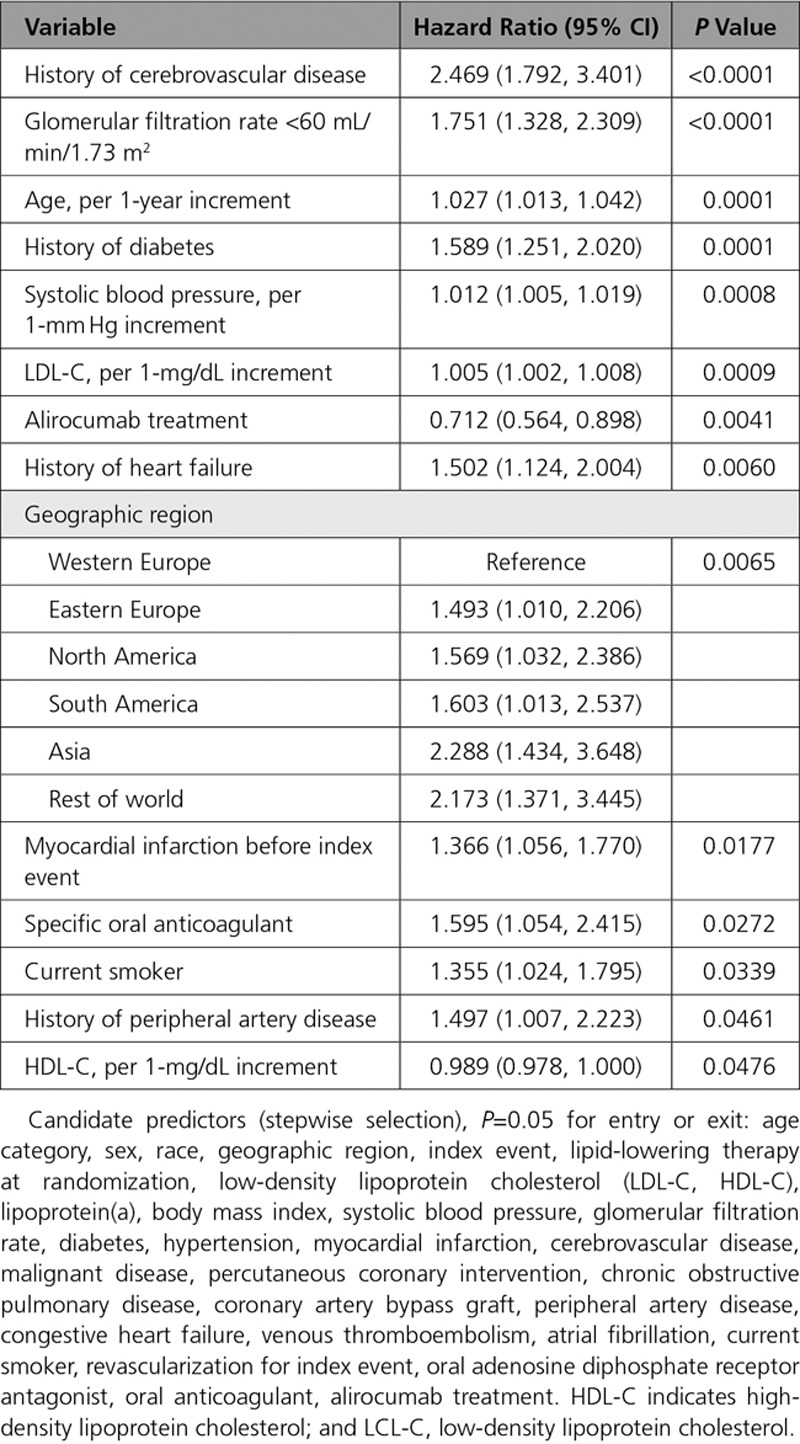
Multivariable Model Predicting Stroke

**Figure 3.**
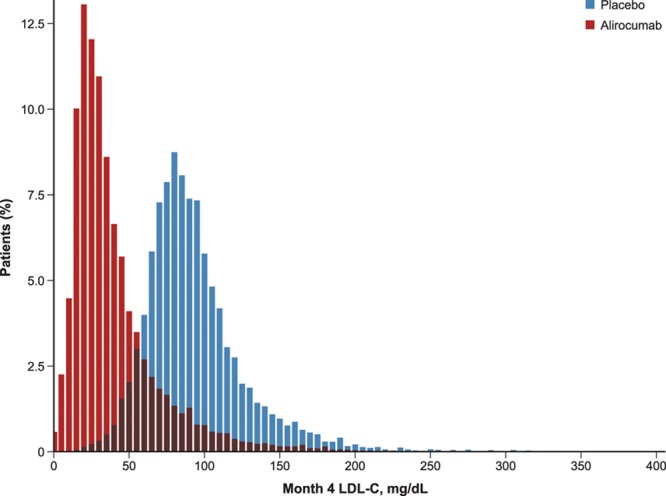
**Month 4 LDL-C by treatment group.** LDL-C indicates low-density lipoprotein cholesterol.

Achieved LDL-C at month 4 by treatment group is shown in Figure 3. Among the 9462 alirocumab-assigned patients, 3397 (35.9%) achieved LDL-C concentrations at month 4 <25 mg/dL, 3749 (39.6%) achieved 25 to <50 mg/dL, 1087 (11.5%) achieved 50 to <70 mg/dL, and 1169 (12.4%) achieved ≥70 mg/dL. Table 3 shows the incidence of hemorrhagic stroke by ordered category of month 4 achieved LDL-C in the alirocumab group. There was no apparent adverse relation between lower achieved LDL-C and incidence of hemorrhagic stroke, with a numerically lower proportion of patients in the lowest categories of achieved LDL-C (ie, <50 mg/dL) experiencing this outcome.

**Table 3.**
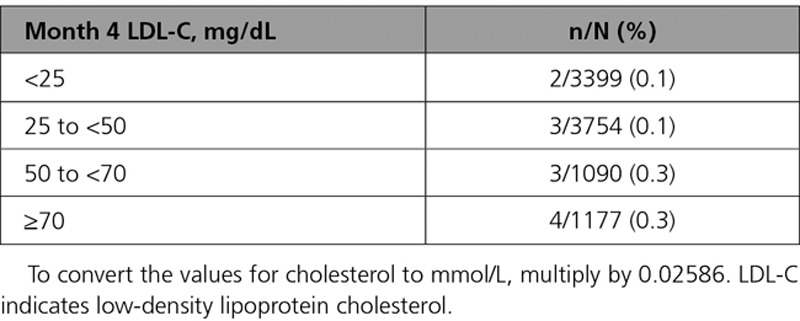
Hemorrhagic Stroke, by Achieved LDL-C Category at 4 Months in Patients Assigned to Alirocumab Treatment

## Discussion

In patients with recent ACS and dyslipidemia despite intensive statin therapy, alirocumab decreased the risk of ischemic stroke without increasing hemorrhagic stroke. Furthermore, risk of hemorrhagic stroke did not depend on achieved LDL-C levels in the alirocumab group.

The treatment effect appeared numerically greater with lower HRs for patients with higher baseline LDL-C, suggesting that patients with a higher risk at baseline have a larger benefit of alirocumab. However, this linear trend was not statistically significant. Furthermore, because qualification for inclusion in the ODYSSEY OUTCOMES trial required an ACS, patients with a history of cerebrovascular disease all had polyvascular disease, which is associated with a high risk of major adverse cardiovascular events and large absolute benefit of alirocumab in reducing such events.^13^ Accordingly, stroke risk was markedly higher in patients with a history of cerebrovascular disease, with a HR of 2.469 (95% CI, 1.792–3.401) in multivariable analysis. However, the treatment effect of alirocumab on stroke was similar in both patients with or without a history of cerebrovascular disease. Therefore, alirocumab is a suitable therapy in patients with recent ACS, irrespective of baseline LDL-C and of history of cerebrovascular disease.

The potential association of very low LDL-C with hemorrhagic stroke risk has been investigated primarily in epidemiologic studies,^14^ but more recently 2 large prospective cohort studies tried to provide clarity on this matter in healthy participants and found increased risks of hemorrhagic stroke with LDL-C <70 mg/dL.^7,15^ The Women’s Health Study in the United States found an adjusted relative risk of 2.17 (95% CI, 1.05–4.48).^15^ The Kailuan study in China reported adjusted HRs of 1.65 (95% CI, 1.32–2.05) for LDL-C 50 to 69 mg/dL and 2.69 (95% CI, 2.03–3.57) for LDL-C <50 mg/dL.^7^ Despite concerns regarding hemorrhagic stroke, our findings that intensive reduction of LDL-C did not cause harm in terms of hemorrhagic stroke reinforces and extends other previous data. The SPARCL trial (Stroke Prevention by Aggressive Reduction in Cholesterol Levels) showed a reduction in overall strokes with high-intensity atorvastatin therapy, despite a small increase in hemorrhagic stroke in 4731 patients with recent stroke or transient ischemic attack.^16^ SPARCL also demonstrated that achieving ≥50% LDL-C lowering was associated with a greater reduction in the risk of ischemic stroke without increasing hemorrhagic stroke and that a higher risk of hemorrhagic stroke was largely in patients with a history of small vessel disease or nonatherothrombotic stroke.^1,17^

Similar conclusions regarding stroke were drawn from the FOURIER trial, investigating the PCSK9 inhibitor evolocumab added to statin therapy in 27 564 patients with stable, established, atherosclerotic cardiovascular disease, including 19.4% with a history of nonhemorrhagic stroke. Evolocumab treatment significantly reduced the risk of ischemic stroke (HR, 0.75 [95% CI, 0.62–0.92]), without a significant effect on hemorrhagic stroke (HR, 1.16 [95% CI, 0.68–1.98]).^10^ Although PCSK9 inhibition may lower LDL-C to levels far below those achieved with statins alone, FOURIER found that lower achieved LDL-C did not increase the risk of hemorrhagic stroke, even when LDL-C levels were <0.5 mmol/L (20 mg/dL).^18^

As most lipid trials have few hemorrhagic stroke events, a meta-analysis was performed recently of randomized trials, including all lipid-lowering trials with statins, ezetimibe, and PCSK9 inhibition.^9^ The investigators found a net benefit of lipid-lowering, with a rate ratio of 0.80 (95% CI, 0.76–0.84) for ischemic stroke and 1.17 (95% CI, 1.03–1.32) for hemorrhagic stroke, for each 1 mmol/L lower LDL-C achieved at about 1 year of follow-up.^9^ Of note, in these studies different types and doses of lipid-lowering therapies were used in patients with or without proven vascular disease in various vascular beds, including coronary artery disease, peripheral artery disease, and cerebrovascular disease. Our results are in line with this meta-analysis in that we found a large benefit of alirocumab in multivariable analysis (HR, 0.712 [95% CI, 0.564–0.898]; *P*=0.0041) and no relationship between very low achieved LDL-C and incidence of hemorrhagic stroke. However, given that only 33 patients had a hemorrhagic stroke, the confidence intervals were large, with an HR in all patients of 0.83 (95% CI, 0.42–1.65). The ongoing Treat Stroke to Target trial of patients with stroke of atherothrombotic origin treated with statins, is testing whether targeting a lower LDL-C level with statins and ezetimibe reduces cardiovascular event rates further, and will also provide additional prospective testing of the safety of that strategy.^19^

### Limitations

Median follow-up was relatively brief, at 2.8 years, and one cannot exclude that the effects of alirocumab on ischemic or particularly hemorrhagic stroke might differ with much longer-term follow-up. Therefore, a relationship of alirocumab treatment to long-term risk of stroke is as yet unknown. A relatively small number of patients had a history of cerebrovascular disease, and therefore the power to detect effects of alirocumab in this subgroup was limited. Only a dedicated randomized controlled trial among individuals with cerebrovascular disease could reliably establish the efficacy and safety in this subgroup. Because blood pressure was generally well controlled in the trial population, the present results may not necessarily apply in populations with uncontrolled blood pressure. In the analyses relating achieved LDL-C levels to subsequent risk of hemorrhagic stroke, patients who achieved lower LDL-C might have other prognostic characteristics placing them at lower risk for this outcome, on average, relative to patients with higher achieved LDL-C. This, in combination with few hemorrhagic strokes after month 4, might in part explain the lack of an observed adverse relationship.

### Conclusions

This analysis of the ODYSSEY OUTCOMES trial shows that in patients with recent ACS and dyslipidemia despite intensive statin therapy, the PCSK9 inhibitor alirocumab decreased the risk of stroke, irrespective of baseline LDL-C and of history of cerebrovascular disease, over a median follow-up of 2.8 years. Furthermore, the present findings indicate that the risk of hemorrhagic stroke did not depend on achieved LDL-C levels in the alirocumab group.

## Acknowledgments

The authors thank the patients, study coordinators, and investigators who participated in this trial. Sophie Rushton-Smith (MedLink Healthcare Communications, London) provided editorial assistance in the preparation of the article (limited to editing for style, referencing, and figure and table editing) and was funded by Fondation Assistance Publique − Hôpitaux de Paris, Paris, France.

## Sources of Funding

This study was supported by Sanofi, Regeneron Pharmaceuticals, Inc.

## Disclosures

Dr Jukema reports research grants from the Netherlands Heart Foundation, the Interuniversity Cardiology Institute of the Netherlands, and the European Commission Seventh Framework Programme; and research support from Amgen, Astellas, AstraZeneca, Daiichi- Sankyo, Lilly, Merck-Schering-Plough, Pfizer, Roche, and Sanofi. Dr Bhatt discloses the following relationships: Advisory Board: Cardax, Cereno Scientific, Elsevier Practice Update Cardiology, Medscape Cardiology, PhaseBio, Regado Biosciences; Board of Directors: Boston VA Research Institute, Society of Cardiovascular Patient Care, TobeSoft; Chair: American Heart Association Quality Oversight Committee; Data Monitoring Committees: Baim Institute for Clinical Research (formerly Harvard Clinical Research Institute, for the PORTICO IDE trial (Portico Re-sheathable Transcatheter Aortic Valve System US IDE Trial), funded by St Jude Medical, now Abbott), Cleveland Clinic (including for the ExCEED trial [CENTERA THV System in Intermediate Risk Patients Who Have Symptomatic, Severe, Calcific, Aortic Stenosis Requiring Aortic Valve Replacement], funded by Edwards), Duke Clinical Research Institute, Mayo Clinic, Mount Sinai School of Medicine (for the ENVISAGE-TAVI AF trial [Edoxaban Compared to Standard Care After Heart Valve Replacement Using a Catheter in Patients With Atrial Fibrillation], funded by Daiichi Sankyo), Population Health Research Institute; Honoraria: American College of Cardiology (Senior Associate Editor, Clinical Trials and News, ACC. org; Vice-Chair, ACC Accreditation Committee), Baim Institute for Clinical Research (formerly Harvard Clinical Research Institute; REDUAL-PCI clinical trial (Evaluation of Dual Therapy With Dabigatran vs. Triple Therapy With Warfarin in Patients With AF That Undergo a PCI With Stenting) steering committee funded by Boehringer Ingelheim; AEGIS-II trial (Study to Investigate CSL112 in Subjects With Acute Coronary Syndrome) executive committee funded by CSL Behring), Belvoir Publications (Editor in Chief, Harvard Heart Letter), Duke Clinical Research Institute (clinical trial steering committees), HMP Global (Editor in Chief, Journal of Invasive Cardiology), Journal of the American College of Cardiology (Guest Editor; Associate Editor), Medtelligence/ReachMD (CME steering committees), Population Health Research Institute (for the COMPASS trial [Rivaroxaban for the Prevention of Major Cardiovascular Events in Coronary or Peripheral Artery Disease] operations committee, publications committee, steering committee, and USA national coleader, funded by Bayer), Slack Publications (Chief Medical Editor, Cardiology Today’s Intervention), Society of Cardiovascular Patient Care (Secretary/Treasurer), WebMD (CME steering committees); Other: Clinical Cardiology (Deputy Editor), NCDR-ACTION Registry Steering Committee (Chair), VA CART Research and Publications Committee (Chair); Research Funding: Abbott, Afimmune, Amarin, Amgen, AstraZeneca, Bayer, Boehringer Ingelheim, Bristol-Myers Squibb, Chiesi, CSL Behring, Eisai, Ethicon, Ferring Pharmaceuticals, Forest Laboratories, Idorsia, Ironwood, Ischemix, Lilly, Medtronic, PhaseBio, Pfizer, Regeneron, Roche, Sanofi-Aventis, Synaptic, The Medicines Company; Royalties: Elsevier (Editor, Cardiovascular Intervention: A Companion to Braunwald’s Heart Disease); Site Co-Investigator: Biotronik, Boston Scientific, St Jude Medical (now Abbott), Svelte; Trustee: American College of Cardiology; Unfunded Research: FlowCo, Fractyl, Merck, Novo Nordisk, PLx Pharma, Takeda. Dr Bittner reports research grants from Amgen, DalCor, Esperion, Sanofi, AstraZeneca, Bayer Healthcare, and The Medicines Company; honoraria from the American College of Cardiology, American Heart Association, and National Lipid Association; and serving as a consultant on advisory boards for Sanofi. Dr Diaz reports research grants from Sanofi, DalCor Pharmaceuticals, Population Health Research Institute, Duke Clinical Research Institute, the TIMI group, Amgen, Cirius, Montreal Health Innovations Coordinating Center and Lepetit and personal fees, as a member of the Executive Steering Committee, from Amgen and Cirius. Dr Drexel reports lecture honoraria, research grants, and advisory boards for: NovoNordisk, Merck, MSD, Pfizer, Sanofi-Aventis, AstraZeneca, Bayer, Takeda, Daiichi-Sankyo, Novartis, Amgen, Boehringer Ingelheim, BMS, Abbott, Janssen-Cilag, and Genericon. Dr Goodman reports research grants from Daiichi-Sankyo, Luitpold Pharmaceuticals, Merck, Novartis, Servier, Regeneron Pharmaceuticals, Sanofi, Amgen, AstraZeneca, Bayer, Boehringer Ingelheim, Bristol-Myers Squibb, CSL Behring, Eli Lilly, Pfizer, and Tenax Therapeutics; honoraria from Bristol-Myers Squibb, Eli Lilly, Esperion, Fenix Group International, Ferring Pharmaceuticals, Merck, Novartis, Pfizer, Servier, Regeneron Pharmaceuticals, Sanofi, Amgen, AstraZeneca, Bayer, and Boehringer Ingelheim; and serving as a consultant or on advisory boards (or both) for AstraZeneca, Boehringer Ingelheim, Bristol-Myers Squibb, Eli Lilly, HLS Therapeutics, Pfizer, Servier, Tenax Therapeutics, Sanofi, Amgen, and Bayer. Dr Kim is an employee of and holds shares in Sanofi. Dr Pordy is an employee of Regeneron Pharmaceuticals, Inc. Dr Reiner has received honoraria from Sanofi. Dr Roe reports research grant funding from Sanofi-Aventis, Astra Zeneca, Patient Centered Outcomes Research Institute, Ferring Pharmaceuticals, Myokardia, Familial Hypercholesterolemia Foundation, and Bayer; and consulting or honoraria from Astra Zeneca, Amgen, Cytokinetics, Eli Lilly, Roche-Genentech, Janssen Pharmaceuticals, Regeneron, Novo Nordisk, Pfizer, Sanofi-Aventis, Signal Path, and Elsevier Publishers. All conflicts of interest are listed at https://www.dcri.org/about-us/conflict-of-interest. Dr Tse has received research funding from Abbott, AstraZeneca, Bayer, Boehringer Ingelheim, Boston Scientific, Daiichi-Sankyo, Pfizer, Sanofi-Aventis, and St Jude Medical (now Abbott). Dr White reports receiving grant support paid to the institution and fees for serving on a steering committee for the ODYSSEY OUTCOMES trial (Evaluation of Cardiovascular Outcomes After an Acute Coronary Syndrome During Treatment With Alirocumab) from Sanofi-Aventis and Regeneron Pharmaceuticals, for the ACCELERATE study (A Study of Evacetrapib in High-Risk Vascular Disease) from Eli Lilly, for the STRENGTH trial (Outcomes Study to Assess Statin Residual Risk Reduction With EpaNova in High CV Risk Patients With Hypertriglyceridemia) from Omthera Pharmaceuticals, for the SPIRE trial (The Evaluation of Bococizumab [PF-04950615; RN 316] in Reducing the Occurrence of Major Cardiovascular Events in High Risk Subjects) from Pfizer USA, for the HEART-FID study (Randomized Placebo-Controlled Trial of FCM as Treatment for Heart Failure With Iron Deficiency) from American Regent; for the CAMELLIA-TIMI study (A Study to Evaluate the Effect of Long-term Treatment With BELVIQ [Lorcaserin HC] on the Incidence of Major Adverse Cardiovascular Events and Conversion to Type 2 Diabetes Mellitus in Obese and Overweight Subjects With Cardiovascular Disease or Multiple Cardiovascular Risk Factors) from Eisai Inc, for the dal-GenE study (Effect of Dalcetrapib vs Placebo on CV Risk in a Genetically Defined Population With a Recent ACS) from DalCor Pharma UK Inc, for the AEGIS-II study from CSL Behring, for the SCORED trial (Effect of Sotagliflozin on Cardiovascular and Renal Events in Patients With Type 2 Diabetes and Moderate Renal Impairment Who Are at Cardiovascular Risk) and the SOLOIST-WHF trial (Effect of Sotagliflozin on Cardiovascular Events in Patients With Type2 Diabetes Post Worsening Heart Failure) from Sanofi-Aventis Australia Pty Ltd, and for the CLEAR Outcomes Study (Evaluation of Major Cardiovascular Events in Patients With, or at High Risk for, Cardiovascular Disease Who Are Statin Intolerant Treated With Bempedoic Acid [ETC-1002] or Placebo) from Esperion Therapeutics Inc. Dr White was on the Advisory Boards for Acetelion, Sirtex and Genentech, Inc. (an affiliate of F. Hoffmann-La Roche Ltd, “Roche”; Lytics Post-PCI Advisory Board at European Society of Cardiology), and received lecture fees from AstraZeneca. Dr Zeiher reports receiving fees for serving on a steering committee for the ODYSSEY OUTCOMES trial from Sanofi, and advisory board and speaker fees from Sanofi, Amgen, Boehringer Ingelheim, Bayer, Novartis, Pfizer, AstraZeneca, and Vifor. Dr Baccara-Dinet is an employee of and holds shares in Sanofi. Dr Szarek reports serving as a consultant or on advisory boards (or both) for CiVi, Resverlogix, Baxter, Esperion, and Regeneron Pharmaceuticals. Dr Schwartz reports research grants to the University of Colorado from Resverlogix, Sanofi, The Medicines Company, and Roche; and is coinventor of pending US patent 14/657192 (“Methods of Reducing Cardiovascular Risk”) assigned in full to the University of Colorado. Dr Steg reports grants and nonfinancial support (cochair of the ODYSSEY OUTCOMES trial; as such he received no personal fees, but his institution has received funding for the time he has devoted to trial coordination, and he has received support for some travel related to trial meetings) from Sanofi; research grants and personal fees from Bayer (Steering Committee MARINER, grant for epidemiological study), Merck (speaker fees, grant for epidemiological studies), Sanofi (cochair of the ODYSSEY OUTCOMES trial; cochair of the SCORED trial; consulting, speaking), Servier (Chair of the CLARIFY registry; grant for epidemiological research), and Amarin (executive steering committee the REDUCE-IT trial [Disease Reduction of Cardiovascular Events With Icosapent Ethyl–Intervention Trial]; consulting); and personal fees from Amgen, Bristol-Myers Squibb, Boehringer Ingelheim, Pfizer, Novartis, Regeneron Pharmaceuticals, Lilly, and AstraZeneca. Dr Steg also has a European application number/patent number, issued on October 26, 2016 (No. 15712241.7), for a method for reducing cardiovascular risk. The other authors report no conflicts.

## Supplementary Material
